# ANCA‐Negative Granulomatosis With Polyangiitis Mimicking Sinusitis and Rhinoscleroma: A Case Report

**DOI:** 10.1155/carm/4144957

**Published:** 2026-02-26

**Authors:** Sergey Gorbunov, Georgiy Polev

**Affiliations:** ^1^ Department of Head and Neck Surgery, Ilyinskaya Hospital, Moscow Oblast, Russia; ^2^ Department of Otorhinolaryngology, Central State Medical Academy, Moscow, Russia

**Keywords:** ANCA-negative, chronic rhinosinusitis, granulomatosis with polyangiitis, rhinoscleroma, saddle nose deformity

## Abstract

**Introduction:**

This case details the diagnostic challenge of ANCA‐negative granulomatosis with polyangiitis (GPA) initially presenting as refractory chronic rhinosinusitis, mimicking recurrent infections, and other granulomatous conditions. It highlights the potential for significant diagnostic delay when serological markers are absent.

**Case Presentation:**

A 65‐year‐old female with recurrent sinusitis underwent multiple antibiotic regimens and endoscopic sinus surgery. Despite this, she developed progressive destructive manifestations over 10 months: nasal septal perforation, saddle nose deformity, keratouveitis with exophthalmos, macrohematuria, and a lacunar cerebellar infarct. Serial microbiology showed various pathogens; histology initially suggested rhinoscleroma. ANCA remained negative.

**Interventions and Outcomes:**

Following the clinical diagnosis of ANCA‐negative GPA, therapy with rituximab and corticosteroids was initiated, leading to significant improvement and sustained remission on maintenance immunosuppression.

**Conclusion:**

This case demonstrates that ANCA‐negative GPA can present as refractory sinonasal disease. Negative serology does not exclude GPA; a high clinical suspicion is warranted in cases with destructive features and systemic progression. Early immunosuppressive treatment is essential to prevent severe organ damage.

## 1. Introduction

Granulomatosis with polyangiitis (GPA), formerly Wegener’s granulomatosis, is a rare autoimmune vasculitis affecting small‐ and medium‐sized vessels, characterized by necrotizing granulomatous inflammation. It commonly involves the upper and lower respiratory tracts and kidneys, with sinonasal manifestations in up to 100% of cases, often mimicking chronic rhinosinusitis [1]. Antineutrophil cytoplasmic antibodies (ANCAs) positivity, particularly cytoplasmic ANCA (c‐ANCA) targeting proteinase 3, is seen in the majority of systemic GPA, while a substantial minority of limited forms and localized disease may be ANCA‐negative; ANCA‐negative disease can account for roughly 10%–20% of cases and is therefore a known diagnostic pitfall [1, 2]. Differential diagnoses include infections (e.g., rhinoscleroma, fungal sinusitis), benign tumors, and malignancies [1, 3]. Early recognition is crucial to prevent destructive complications like septal perforation or orbital involvement.

## 2. Case Presentation

**Figure 1:**
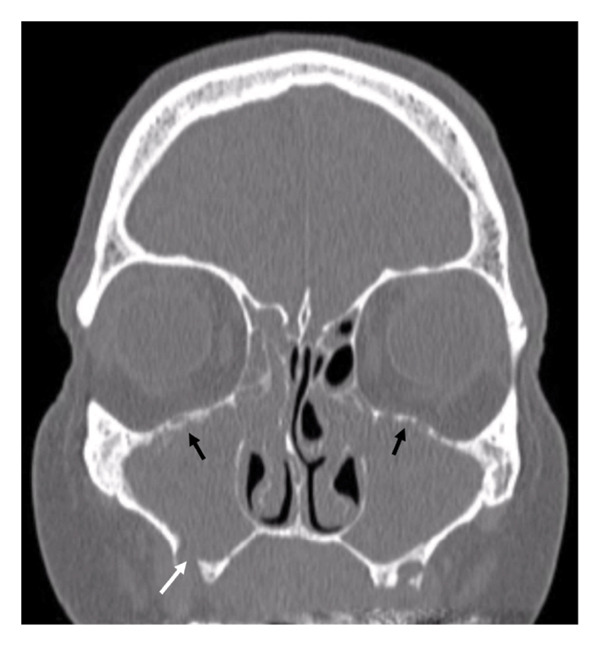
Computed tomography of the paranasal sinuses prior to surgery. The white arrow indicates the tooth extraction site with a bone defect. Black arrows indicate areas of osteitis in the superior walls of the maxillary sinuses.

A 65‐year‐old woman presented in January 2024 with acute rhinosinusitis, treated with penicillins. In February 2024, facial pain recurred, leading to a diagnosis of odontogenic maxillary sinusitis; teeth were extracted, and cephalosporins were administered, but oroantral communication healed slowly. Symptoms relapsed in May 2024, with computed tomography (CT) showing inflammation in maxillary and right frontal sinus (Figure 1); ciprofloxacin was prescribed for 2 weeks.

**Figure 2:**
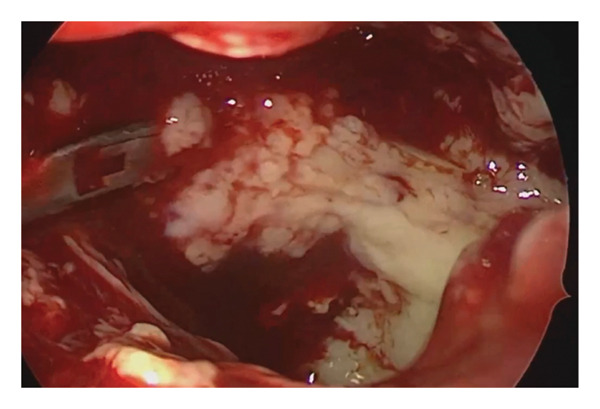
Endoscopic view of the left maxillary sinus (70‐degree endoscope) during sinus surgery showing copious purulent discharge.

In June 2024, endoscopic bilateral maxillary sinusotomy and right‐sided Draf IIa frontal sinusotomy were performed (Figure 2). Histology confirmed inflammation, and intraoperative culture identified *Staphylococcus lugdunensis* (10^2^ CFU/mL). Postoperative endoscopy revealed crusting and purulent plaques in both maxillary sinuses. Nasal swabs in July 2024 showed *Klebsiella oxytoca* (10^3^ CFU/mL) and *Staphylococcus epidermidis* (10^3^ CFU/mL). By August 2024, *Staphylococcus aureus* (10^6^ CFU/mL, later methicillin‐resistant *Staphylococcus aureus* (MRSA) at 10^3^–10^4^ CFU/mL) predominated, alongside *Alternaria alternata* and yeast cells on fluorescent microscopy (no *Candida* growth). Mycobacterial PCR was negative.

**Figure 3:**
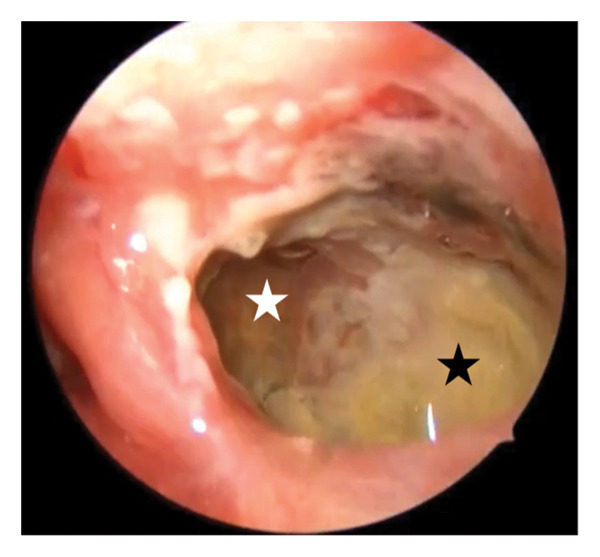
Endoscopic view of the left maxillary sinus 4 months postoperative (70‐degree endoscope). Yellowish plaques within the sinus cavity (black asterisk) with areas of intact mucosa (white asterisk).

From July to September 2024, symptoms persisted with white/yellowish plaques on nasal endoscopy (Figure 3). Blood tests showed elevated erythrocyte sedimentation rate (ESR) (53–81 mm/h), mild anemia (hemoglobin 11.0–12.8 g/dL), and occasional C‐reactive protein (CRP) elevations (up to 9.5 mg/L); other parameters (liver enzymes, renal function, immunoglobulins, and eosinophils) were largely normal. Treatment included trimethoprim/sulfamethoxazole 480 mg three times daily for 14 days, resulting in significant improvement; a 10‐day course of linezolid 600 mg twice daily with slight improvement; and clindamycin 150 mg four times daily for 10 days without significant effect. Subsequently, a 1‐month regimen of trimethoprim/sulfamethoxazole 960 mg twice daily combined with fluconazole 200 mg once daily was administered, achieving marked clinical improvement. Sinus lavage with chlorhexidine was also performed during this period.

In November 2024, cytology indicated marked inflammation without malignancy. Histopathology revealed extensive areas of necrosis surrounded by spindle‐cell proliferations composed of mixed small CD3‐positive T lymphocytes and CD20‐positive B lymphocytes, foamy macrophages containing intracytoplasmic Periodic acid–Schiff (PAS)‐positive material, and neutrophils. Immunohistochemistry showed preserved cytokeratin (AE1/AE3) expression within normal limits, low proliferative activity (Ki‐67 reminder index ∼10%), negative CD56, desmin, Epstein–Barr virus, and absence of aberrant lymphoid marker expression, thereby excluding epithelial malignancy, NK/T‐cell lymphoma, and other lymphoproliferative disorders. Grocott staining did not reveal fungal hyphae or spores. Based on these findings, infectious and neoplastic processes were considered unlikely, and a diagnosis of rhinoscleroma was initially favored. A subsequent reference histopathological review confirmed a dense chronic inflammatory infiltrate extending through the lamina propria, dominated by large foamy histiocytes (Mikulicz cells), some S‐100 positive, with abundant plasma cells, focal necrosis, fibrosis, and PAS‐positive material within histiocytes. The immunophenotype and morphology were consistent with rhinoscleroma (specific granulomatous inflammation). Chest CT was normal (postinflammatory changes only). Treatment included doxycycline (100 mg BID for 10 days), linezolid (600 mg BID for 3 weeks), rifampicin (150 mg TID for 4 weeks), and maxillary sinuses lavage with Prontosan.

Early December 2024, CT showed hyperplastic pansinusitis, bilateral otitis media, and mastoid inflammation without bone destruction. ANCA testing was performed by indirect immunofluorescence (IFA), showing ANCA IgG < 1:40 (cytoplasmic pattern); confirmatory enzyme‐linked immunosorbent assays (ELISA) for antiproteinase‐3 (PR3) IgG and anti‐myeloperoxidase (MPO) IgG were negative. Additional autoantibody screening was negative, including SS‐A/Ro‐52, SS‐A/Ro‐60, SS‐B/La, RNP/Sm, Sm, Scl‐70, Jo‐1, dsDNA, nucleosomes, histones, Pm‐Scl, AMA‐M2, PCNA, centromere‐B, and ribosomal *P* protein. Serological testing for including human immunodeficiency virus (HIV), viral hepatitis, and syphilis was negative; immunological testing demonstrated normal IgA, IgM, IgG, IgG4, and complement C3 and C4. ESR peaked at 96 mm/h, with anemia (hemoglobin 11.4 g/dL) and leukocytes 9.41 × 10^3^/μL; eosinophil counts remained within the reference range. New symptoms included dry eyes, photophobia, and ear fullness.

**Figure 4:**
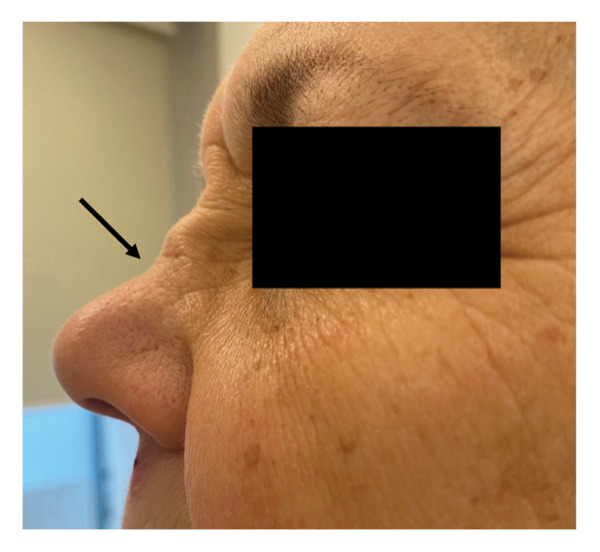
External view of nose demonstrating saddle‐nose deformity (black arrow).

**Figure 5:**
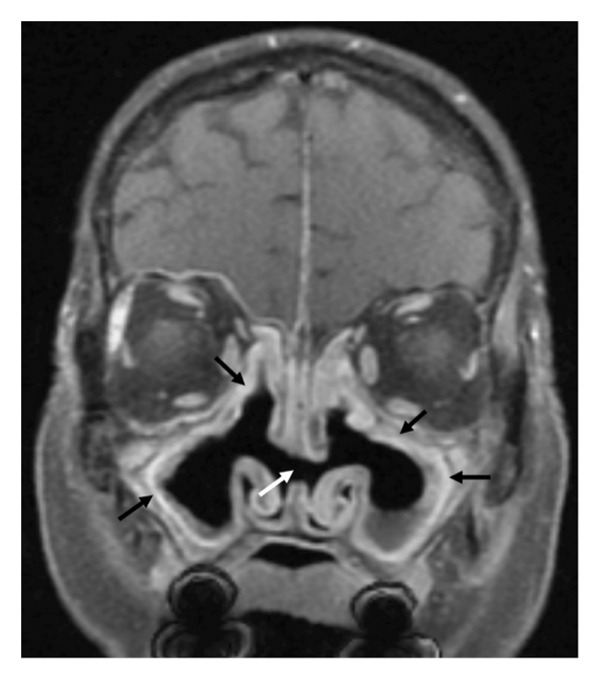
Magnetic resonance imaging of the head. Postoperative changes in the paranasal sinuses (black arrows) and nasal septal perforation (white arrow).

By late December 2024, rapid progression occurred: keratouveitis, right‐sided exophthalmos, macrohematuria with proteinuria of 0.281 g/L, detected on routine urinalysis, and red blood cells > 100/HPF, slight dizziness, saddle‐nose deformity, and nasal septal perforation (Figures 4 and 5). 24‐h urinary protein excretion was not assessed. Renal biopsy was not performed due to mild proteinuria, preserved renal function, and high clinical probability of systemic vasculitis requiring urgent immunosuppressive therapy.

Magnetic resonance imaging confirmed a lacunar ischemic infarct (6–6.5 mm) in the left cerebellar hemisphere, orbital edema, enlarged right lacrimal gland, pansinusitis, and postsurgical changes (Figure 5). Coagulation was abnormal (prolonged prothrombin time/international normalized ratio [PT/INR], activated partial thromboplastin time [APTT], with elevated D‐dimer (817 ng/mL) and CRP (45.6 mg/L)). The patient was admitted to the rheumatology department for further management. ANCA‐negative GPA was clinically diagnosed based on sinonasal destruction, orbital involvement, and hematuria. Treatment with prednisolone and rituximab was initiated.

By July 2025, the patient remained under planned follow‐up on maintenance therapy including mycophenolate and low‐dose prednisone, with admission for a scheduled rituximab infusion. Laboratory tests and urinalysis were within normal limits, infectious screening was negative, and imaging showed persistent pansinusitis without pulmonary involvement. Overall, the patient demonstrated sustained clinical and laboratory improvement with stabilization of renal function.

## 3. Discussion

This case exemplifies the diagnostic complexity of ANCA‐negative GPA, where initial presentations mimic infectious sinusitis, leading to repeated antibiotics and surgery. Similar single‐center reports and case series document that limited or localized GPA can present solely with chronic rhinosinusitis or otologic complaints and be initially misdiagnosed as refractory infection [4–6]. Histopathology suggesting rhinoscleroma (foamy macrophages and PAS‐positive material) created a “red herring,” as rhinoscleroma typically lacks systemic features like hematuria or stroke. Negative ANCA did not exclude GPA, as limited otolaryngological disease often lacks serological markers [2]. Moreover, ANCA‐negative GPA may be associated with autoantibodies that are undetectable by conventional methods or of unknown specificity [7].

ENT‐limited and ANCA‐negative presentations of GPA are well‐documented. In a retrospective otolaryngology cohort of 143 patients, 18.2% were ANCA‐negative at presentation, most commonly with sinonasal disease (73.9%), only 12% of patients had diagnostic biopsy findings, and only 17.4% had renal involvement [6]. Renal involvement in ANCA‐negative GPA may be mild initially, manifesting as hematuria and low‐level proteinuria without overt renal failure, and renal biopsy may not always be performed at first presentation. Diagnostic delays in seronegative cases reach 11 years [6].

Histopathology in limited or ANCA‐negative GPA is often nonspecific and may not reveal classic vasculitis, especially in superficial mucosal biopsies, necessitating careful clinicopathological correlation rather than reliance on isolated serological or histological markers [8].

Rapid multisystem progression (septal perforation, saddle nose, orbital edema, renal involvement, and cerebellar infarct) aligned with GPA’s vasculitic nature and the sinonasal imaging patterns described in recent series (mucosal thickening, bone changes, and orbital invasion possible on CT) [3]. Rituximab and steroids, used for induction/maintenance in GPA, yielded improvement in this patient and are supported by treatment literature and reviews [1].

## 4. Conclusion

ANCA‐negative GPA can masquerade as chronic sinusitis or infections like rhinoscleroma, delaying diagnosis. Clinicians must consider vasculitis in persistent cases with destructive features, as negative serology does not preclude GPA. Early immunosuppressive therapy can prevent severe complications.

## Author Contributions

Sergey Gorbunov and Georgiy Polev: clinical care and manuscript drafting, and literature review and editing.

## Funding

No funding was received for this manuscript.

## Disclosure

All authors read and approved the final manuscript.

## Ethics Statement

All procedures involving humans were performed in accordance with the ethical standards of the Ilyinskaya Hospital and the Helsinki Declaration. Informed consent was obtained from all participants.

## Conflicts of Interest

The authors declare no conflicts of interest.
